# Towards precision medicine: interrogating the human genome to identify drug pathways associated with potentially functional, population-differentiated polymorphisms

**DOI:** 10.1038/s41397-019-0096-y

**Published:** 2019-10-03

**Authors:** Maulana Bachtiar, Brandon Nick Sern Ooi, Jingbo Wang, Yu Jin, Tin Wee Tan, Samuel S. Chong, Caroline G. L. Lee

**Affiliations:** 1 Department of Biochemistry, Yong Loo Lin School of Medicine, National University of Singapore, Singapore, Singapore; 2 Division of Medical Sciences, Humphrey Oei Institute of Cancer Research, National Cancer Centre Singapore, Singapore, Singapore; 3 National Supercomputing Centre Singapore, Singapore, Singapore; 4 Department of Pediatrics, Yong Loo Lin School of Medicine, National University of Singapore, Singapore, Singapore; 5 Cancer & Stem Cell Biology Programme, Duke-NUS Graduate Medical School, Singapore, Singapore

**Keywords:** Personalized medicine, Pharmacogenomics

## Abstract

Drug response variations amongst different individuals/populations are influenced by several factors including allele frequency differences of single nucleotide polymorphisms (SNPs) that functionally affect drug-response genes. Here, we aim to identify drugs that potentially exhibit population differences in response using SNP data mining and analytics. Ninety-one pairwise-comparisons of >22,000,000 SNPs from the 1000 Genomes Project, across 14 different populations, were performed to identify ‘population-differentiated’ SNPs (pdSNPs). Potentially-functional pdSNPs (pf-pdSNPs) were then selected, mapped into genes, and integrated with drug–gene databases to identify ‘population-differentiated’ drugs enriched with genes carrying pf-pdSNPs. 1191 clinically-approved drugs were found to be significantly enriched (*Z* > 2.58) with genes carrying SNPs that were differentiated in one or more population-pair comparisons. Thirteen drugs were found to be enriched with such differentiated genes across all 91 population-pairs. Notably, 82% of drugs, which were previously reported in the literature to exhibit population differences in response were also found by this method to contain a significant enrichment of population specific differentiated SNPs. Furthermore, drugs with genetic testing labels, or those suspected to cause adverse reactions, contained a significantly larger number (*P* < 0.01) of population-pairs with enriched pf-pdSNPs compared with those without these labels. This pioneering effort at harnessing big-data pharmacogenomics to identify ‘population differentiated’ drugs could help to facilitate data-driven decision-making for a more personalized medicine.

## Introduction

Different individuals with the same disease respond differently to the same drug treatment, and some may experience adverse drug reaction (ADR) [1]. According to the 2007–2009 US FDA Adverse Event Reporting System (FAERS), there were 70,187 ADR cases. The Singapore Health Science Authority (HSA), indicated that from 2007 to 2009 there were 8137 ADR cases in Singapore where many of these drugs were imported from the United States. ADR, the sixth major cause of death in the USA, is a serious public health problem that can result in patient’s discomfort, morbidity, and even mortality. ADR also incurs a huge economic burden due to its related treatment and hospitalization [2, 3]. Although individually tailored treatment is highly desirable to avoid ADR [4, 5], it is not always practical because of the high cost associated with developing such personalized therapy, as well as unavailability of complete information on the true existence of a drug–gene interaction. As such, this has deterred many pharmaceutical companies from adopting this approach in drug development [6].

Differences in drug response/ADR occurrence in different ethnic/racial populations, also referred as ‘pharmacoethnicity’, have been widely reported [7–9]. However, currently, drugs or their dosages are often prescribed to patients of different ethnicities without much consideration to the differences in genetics between the different populations [9, 10]. Although the use of ethnicity/race as a step toward a more personalized treatment has met with opposition and challenges [7], it is a useful proxy to facilitate tailored drug treatment to specific groups of individuals who share greater genetic similarity with each other than with other population groups [11]. Differences in drug response between the European and African/East Asians populations are the most frequently reported, likely due to drugs being primarily tested in the USA/Europe and marketed in other regions [12, 13]. Several common drugs, including abacavir [14], carbamazepine [15, 16], cyclosporine [17], 5-fluorouracil [18–21], tacrolimus [22, 23], vincristine [24], and warfarin [25], have been reported to show population differences in their responses [7].

Because environmental and genetic factors can influence drug response or ADR occurence, elucidating the genetic basis underlying these responses may help in enhancing their prediction [6, 9, 26–31]. For instance, Renbarger et al. showed that African Americans were not as susceptible to vincristine related toxicities as that of Caucasians [24]. This is consistent with the observation of major difference in the CYP3A5*3 allele frequency between Caucasians and African Americans. We hypothesize that genetic factors play a significant role in determining population differences in drug response. Furthermore, these differences are likely caused by differences in allele frequencies of single nucleotide polymorphisms (SNPs) that functionally affect the expression or function of genes in the drug pathway. With the advent of comprehensive genomic and drug–gene knowledge databases, as well as ‘big data’ analytics, we can capitalize on these genetic differences to develop tools to decode important population differentiation patterns that are linked to drug response. Although its application in other fields are emerging, a big-data approach is less explored in pharmacogenomics due to several challenges including its requirement for a multidisciplinary approach and complex data integration and interpretation [32, 33].

This study aims to employ big-data pharmacogenomics to decode important population differentiation patterns in human genes linked to drug response. New insights gleaned from this study can facilitate the selection of candidate potentially functional, population-differentiated SNPs (pf-pdSNPs), and genes in drug response and guide future decision making concerning drug treatment options for specific ethnic populations.

## Materials and methods

### Overview of PGx analytics method

**Fig. 1:**
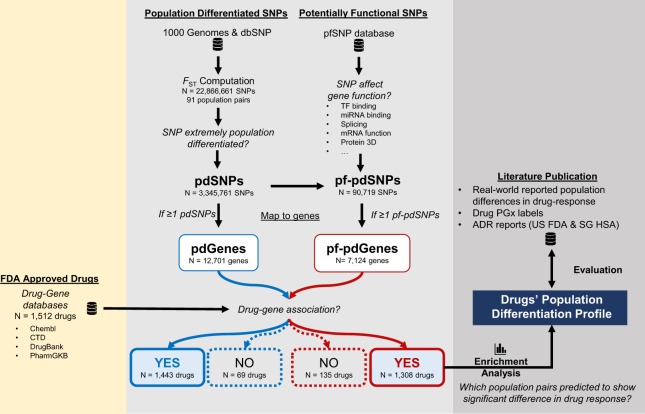
Big-data and deep analytics approach to identify drugs associated with genes carrying SNPs that are differentiated between different populations. *F*_ST_ statistics were determined for all SNPs from the 1000 Genome Project and dbSNP. SNPs with *F*_ST_ statistics in the top 1% of all SNPs in each population pair comparison were regarded as pdSNPs. pdSNPs were then queried against the pfSNP database (http://pfs.nus.edu.sg/) to identify potentially functional pf-pdSNPs. Genic pf-pdSNPs were then mapped to their corresponding genes and genes containing at least one pf-pdSNPs were named pf-pdGene. Four databases (CTD, Chembl, DrugBank, and PharmGKB) were employed to identify genes associated with drugs/drug pathways. Multiple random samplings-based statistical analyses was performed to identify drugs that are enriched with pf-pdGenes (enrichment *Z*-score > 2.58). The robustness of the algorithm was evaluated for its capability to detect such enrichment in drugs previously reported with a real-world population differences in response. We also determined if drugs with pharmacogenetics (PGx) warning labels or adverse drug reaction (ADR) reports are associated with population differentiation

To facilitate the identification of drugs that are predicted to exhibit significant population differences in response between a pair of population examined, we employed a novel ‘PGx analytics’ method as detailed in Fig. 1. The approach involves evaluating each SNP based on two properties: (1) whether the allele frequency of the SNP in one population is significantly different from the frequency in another population and (2) whether the SNP is predicted to be potentially functional affecting either gene/protein expression or activity. SNPs that fulfill either the first criteria alone pdSNP, or both criteria pf-pdSNPs were mapped to their corresponding genes. Genes containing these pf-pdSNPs were then mapped to drug pathways using publicly available drug–gene databases. Multiple random samplings-based statistical analyses were subsequently performed to identify drugs that have an enriched representation of genes carrying pf-pdSNPs. This approach was then evaluated for concordance with literature reported real-world occurrences of population difference in drug response. In addition, the relationship between drugs enriched with genes carrying pf-pdSNPs and PGx warning labels or adverse reaction reports was investigated to provide further evidence of the utility of this method.

### Identification of potentially functional, population differentiated SNPs

A big-data approach was employed to identify drugs enriched with genes carrying pfSNPs that exhibit significant population differentiation (Fig. 1). SNPs with significant population differentiation were identified using data from the 1000 Genomes Project comprising a total of 1029 unrelated individuals representing 14 different populations, including 59 ASW (African ancestries from Southwest United States); 79 LWK (Luhya individuals in Kenya); 86 YRI (Yoruba individuals in Nigeria); 97 CHB (Han Chinese in Beijing); 100 CHS (Han individuals in Southern China); 89 JPT (Japanese individuals in Tokyo); 60 CLM (Columbian in Medellin); 58 MXL (Mexicans in Los Angeles USA); 55 PUR (Puerto Rican in Puerto Rico); 54 CEU (Northern and Western European ancestries in Utah USA); 93 FIN (Finish in Finland); 87 GBR (British in England and Scotland); 98 TSI (Toscani in Italy); and 14 IBS (Iberian in Spain) [34].

To identify SNPs which are associated with significant population variations, allele frequencies of SNPs data-mined from the 1000 Genomes Project [34] were calculated using VCF tools (version 0.1.9). Pairwise *F*_ST_ statistics [35, 36] were computed in the ‘R’ environment for each SNP with each of the 14 population-pair comparisons, resulting in 91 different population-pair *F*_ST_ scores for each SNP. pd using pairwise *F*_ST_ was determined on 71.56% (22,866,661) of the total SNPs from the 1000 Genomes Project but was not computed for the other 28.44% of SNPs as some SNPs were only observed in a single population, while others had variant call errors. The *F*_ST_ statistic is a measure of the proportion of genetic variance found within a population relative to the genetic variance found in both populations and is often defined as: [37]

$$F_{\mathrm{ST}} = \frac{{H_{\rm{T}} - H_{\rm{S}}}}{{H_{\rm{T}}}}$$

For polymorphic biallelic markers where *M* is the mean frequency of the more frequent allele across *K* subpopulations, *p*_k_ is the frequency of the allele in subpopulation *k*, *n*_k_ is the size of subpopulation k and *N* is the sum of subpopulation sizes:

$$H_{\mathrm{S}} = 1 - \frac{1}{N}\mathop {\sum }\limits_{k = 1}^K n_k[p_k^2 + \left( {1 - p_k} \right)^2]$$

and

$$H_{\mathrm{T}} = 1 - [M^2 + \left( {1 - M} \right)^2]$$

In this study, as only pairwise comparisons were made, *K* was set to 2. Between two populations, a SNP is regarded as a population differentiated SNP or pdSNP if its *F*_ST_ score is amongst the top 1% of all the *F*_ST_ scores in the respective pairwise population comparison. This allowed us to extract SNPs that are considered to be extremely population differentiated between two populations by considering those positioned at the top one percentile with respect to the *F*_ST_ scores distribution.

SNPs were mapped to functional gene regions and categorized based on their location according to the NCBI dbSNP (build 137) [38]. In the coding region (i.e., exons), amino acid-substituting SNPs are classified as nonsynonymous (nsSNPs), whereas the silent or nonamino acid-substituting SNPs are referred as synonymous (sSNPs). For SNPs in noncoding regions, the following classifications were applied: promoter for SNPs residing within 5.5 Kb upstream of a gene transcription start site; intronic for SNPs residing in introns; as well as 5′ UTR and 3′ UTR for SNPs residing in the 5′ or 3′ terminal of mRNA untranslated regions.

pdSNPs, which were predicted/evaluated to be potentially functional were named potentially functional (pf) population differentiated (pd) SNPs or pf-pdSNPs. The pfSNP (http://pfs.nus.edu.sg/) resource [39] developed by our laboratory was employed to evaluate the potential functionality of the pdSNPs (Supplementary Table 1). pfSNPs are defined as SNPs, where a single nucleotide change is predicted to either alter the expression, structure, function, or activity of the associated gene/protein or their isoform, or that reside within regions that are genetically determined to be under natural selection forces. For coding SNPs, we evaluated if they reside within important protein domains/functional regions, potentially altering important protein modification sites (e.g., phosphorylation sites) [40], or are predicted to alter nonsense-mediated decay or exonic splice enhancer/silencer sites [41, 42]. sSNPs within the coding region were further evaluated for significant codon usage bias as this may potentially influence translational speed and structure/function of the protein [43, 44], while nsSNPs were selected if they were predicted to be deleterious [45–48].

For noncoding SNPs, those residing in the promoter/5′ UTR regions were evaluated to see if they alter transcription factor binding sites, while those in 3′ UTR were selected if they reside within 3′ UTR conserved regions [49], as they may have functional consequences [50] or alter miRNA binding sites [51–53]. Noncoding SNPs in introns were selected if they alter splice sites [54] or intronic splice regulatory elements [55]. The pd-SNPs and pf-pdSNPs were then mapped on to genes in the following way. A pdGene is a gene, which carries at least one pdSNP, while a gene containing at least one pf-pdSNP is regarded as a pf-pdGene. Supplementary Table 2 contains an explanation of all the abbreviations used in the paper.

### Enrichment analyses of pf-pdGenes in drug pathways for identification of drugs with population differentiated response

To identify drugs (pf-pdDrugs) enriched with genes carrying pf-pdSNPs, we integrated four major literature-backed drug–gene databases (PharmGKB [56], Chembl [57], Comparative Toxicogenomics Database (CTD) [58], and Drug Bank [59]) to obtain drug–gene information from 10,902 unique drugs/compounds (Supplementary Fig. 1). The identification of genes in the pathway of the drugs is based on scientific, peer-reviewed literature evidence curated by these four databases. An example of a few genes documented to be associated with the drug statin is shown in Supplementary Table 3. Through the integration of the FDA approved drugs/compounds with these four drug–gene databases, gene information for 1512 FDA-approved drugs were obtained for this study. These drugs were then evaluated for enrichment of pf-pdGenes in their drug pathway as follows.

The population-pair specific enrichment *Z*-score of each drug was obtained by performing 10,000 sampling iterations involving random genes that are of a similar size range to the genes in that drug pathway. For each drug random sampling set, the proportion of pf-pdGenes found in the random sample was recorded. These 10,000 iterations would yield an empirical distribution specific to the drug and population-pair in question. The population-pair *Z*-scores of the drug will signify enrichment of the observed proportion of pf-pdGenes in the drug pathway relative to the empirical distribution generated in the random sampling, which can be calculated with the following equation.

$$Z\;score = \frac{{p_{{\mathrm{pf}} - {\mathrm{pdGenes}}} - \bar P_{{\mathrm{pf}} - {\mathrm{pdGenes}}}}}{{eSD_{{\mathrm{pf}} - {\mathrm{pdGenes}}}}},$$

where:

p_pf-pdGenes_ = observed proportion of pf-pdGenes in the drug pathway for the specific population-pair

${\bar{\mathrm{P}}}_{{\mathrm{pf}} - {\mathrm{pdGenes}}}$ = mean proportion of pf-pdGenes in the empirical distribution of the respective drug for the specific population-pair

eSD_pf-pdGenes_ = standard deviation of empirical distribution of the respective drug for the specific population-pair

A drug that is significantly enriched with pf-pdGenes for a population-pair has a *Z*-score of >2.58 or is within the top 0.5 percentile of the respective empirical distribution. On the other hand, a drug that is not enriched in pf-pdGenes for that population-pair has a low enrichment *Z*-score for that population-pair. The availability of SNP and gene information from 14 different populations in our database resulted in each drug having up to 91 population-pair *Z*-scores. This enabled us to identify the specific pair of populations with an enrichment of pf-pdGenes in a particular drug pathway.

### Evaluating the performance of the algorithm

The performance of the algorithm was evaluated in terms of whether it can appropriately detect population differentiation patterns in drugs that have been previously reported [7] to show population differences in response. This could provide an initial gauge on the potential capability and real-world relevance of our approach. Only drugs previously reported to be associated with population differences in response and with available population-pair enrichment *Z*-score were included in this literature-based evaluation. Supplementary Table 4 details the publications of the drugs that were reported to be population differentiated. It includes information about the actual population pairs reported and the population pair from our database that was most similar to the one reported. The accuracy of our method was evaluated by comparing the concordance between the drugs that pass the *Z*-score threshold from our algorithm with the drugs found from the literature. The maximum and minimum *F*_ST_ scores specific to the reported drug for each population pair were also determined.

### Classification and ranking of population differentiated drugs by drug classes/disease conditions

The Anatomical Therapeutic Chemical (ATC) Classification System by the World Health Organization (WHO) (http://www.whocc.no/) or manual curation was employed to categorize drugs found to be population differentiated by our algorithm into their respective 414 drug classes, while the CTD database was used to categorize these drugs based on their associated 2783 disease conditions. Only drug classes (*n* = 134) or disease groups (*n* = 1775) containing three or more drug members were included in our analyses. We then ranked the drug classes/diseases groups by multiplying the mode of all population pair *Z*-scores (most common *Z*-score) for each drug by the observed number of population pairs exhibiting significant population differentiation for that drug. This value for each drug in the drug class is, then summed and normalized against the number of constituent drugs within each drug class/disease group. After obtaining the top 30 drug classes/disease groups, information about each individual drug’s number of enriched population pairs was extracted and presented in graphical form.

### Analyses of pharmacogenetic labels and ADR reports

We further assessed whether drugs with existing pharmacogenetic (PGx) warning labels or ADR reports were associated with the number of significantly enriched population pairs. Drugs with PGx warning labels were obtained from PharmGKB [56], which contains information issued by the US Food and Drug Administration (FDA), European Medicines Agency (EMA), Japan’s Pharmaceuticals and Medical Devices Agency (PMDA), and Health Canada (Santé Canada) (HCSC). The four categories of drugs labels or PharmGKB ‘PGx Levels’ used in this study were ‘Genetic testing required’, ‘Genetic testing recommended’, ‘Actionable PGx’, and ‘Informative PGX’. The definitions of these labels can be found at https://www.pharmgkb.org/page/drugLabelLegend.

The ADR reports summary was obtained from publicly available quarterly reports of the US FAERS database (2007–2009) (https://www.fda.gov/Drugs/GuidanceComplianceRegulatoryInformation/Surveillance/AdverseDrugEffects/) and from Pharmacovigilance Branch at the Singapore Health Science Authority (HSA) (http://www.hsa.gov.sg/content/hsa/en/Health_Products_Regulation/Safety_Information_and_Product_Recalls/Report_Adverse_Events_related_to_health_products.html) (2007–2009). Both databases are based on voluntary reporting of suspected ADR, which can be directly submitted by healthcare professionals and consumers or through mandatory reporting from drug manufacturers.

Cumulative distribution function (CDF) plots of the number of significant population-pairs against the fraction of drugs with PGx labels/adverse drug reactions were constructed in R. Bar plots of the average number of population pairs showing significant genomic differentiation across the different PGx labelled drug groups/ADR groups were also constructed in R and statistical significance assessed using the two-sided Student's *t*-test. There was a total of 373 drugs with no ADR reports, 978 drugs with ADR reports, 966 drugs with ADR reports from US, and 391 drugs with reports from Singapore. For the PGx labels, there were 1206 drugs with no PGx labels, 124 drugs with ‘Genetic Testing Recommended’, and 22 drugs with ‘Genetic Testing Required’. All groups had a similar variance. The incidence rates and population pair profiles of the top 20 drugs with the highest ADR rates in Singapore and in the USA were also compared.

## Results

### Deep analytics identifies drugs enriched with genes carrying SNPs that display significant population differentiation

Over 3,000,000 SNPs were identified to be significantly population-differentiated (pdSNPs, *N* = 3,345,761), while ~2.7% of these were also predicted to be potentially functional (pf-pdSNPs, *N* = 90,719) (Fig. 1). Sixty-nine FDA-approved drugs/compounds did not contain a single significantly pdSNP (Supplementary Table 5). 1443 drugs were associated with genes carrying at least one pdSNP, while 1308 of these drugs were associated with genes that carry at least one pf-pdSNP (Supplementary Fig. 2, lower panel).

**Fig. 2:**
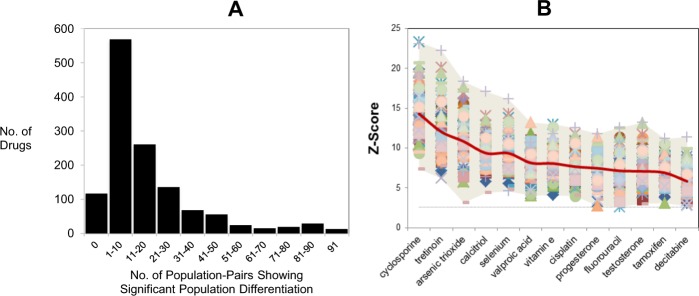
Drugs associated with population-differentiated genes. **a** Distribution of the number of drugs that are enriched (*Z* > 2.58) by pf-pdGenes exhibiting population differentiation patterns across the respective number of population pairs. **b** Drugs enriched by genes (*Z* > 2.58) that are population differentiated in all 91 population-pairs examined, sorted by the average *Z*-score. Each data point corresponds to a specific population pair, with each drug having *Z*-scores across 91 population-pairs. The dotted horizontal line signifies *Z* = 2.58, the threshold *Z*-score for the significant enrichment of pf-pdGenes in the respective population-pair. Red line indicates the average *Z*-score across the drugs, while the shaded area indicates the minimum and maximum value (the range) of the *Z*-scores of the respective drug

As drugs/compounds significantly enriched in pf-pdGenes may have a stronger genetic basis to account for population differences in response, enrichment analyses were performed on the 1308 drugs associated with pf-pdGenes (Fig. 1). Figure 2a shows the distribution of the number of drugs (pf-pdDrugs) significantly enriched with population-differentiated genes (*Z* > 2.58) across the various number of population pairs. Although 1308 drugs were associated with at least one pf-pdGene, 117 of these were not significantly enriched with pf-pdGenes in any of the population-pairs examined (Fig. 2a). The majority of the pf-pdDrugs were observed to be enriched with pf-pdGenes in ~1–10 population pairs (Fig. 2a), while 13 pf-pdDrugs, including common immunosuppressant and anticancer drugs such as cyclosporine, fluorouracil, tamoxifen, and decitabine, were enriched with pf-pdGenes (*Z* > 2.58) in all 91 population-pairs examined (Fig. 2b). One hundred and thirty-three drugs were enriched with pf-pdGenes (*Z* > 2.58) in >45 out of all 91 population-pairs examined (>50% of the pairwise comparisons), while 1191 drugs were enriched with pf-pdGenes (*Z* > 2.58) in at least one of the population-pairs studied. The *Z*-scores for all the pf-pdDrugs across all 91 population pairs are interactively presented at http://rpubs.com/jinyu1104/462707.

### Pharmacogenomics data analytics was capable of identifying drugs that were previously reported to have a population differentiated response

To evaluate the potential capability and real-world relevance of our pharmacogenomics data analytics workflow, we examined commonly prescribed drugs that were previously reported to show differences in response between different populations (see review [7] and Supplementary Table 4). The 11 drugs commonly implicated with real-world reported population differences in response include cyclosporine, fluorouracil, doxorubicin, nicotine, vincristine, estrogens, codeine, gefitinib, diazepam, warfarin, and clomipramine.

**Fig. 3:**
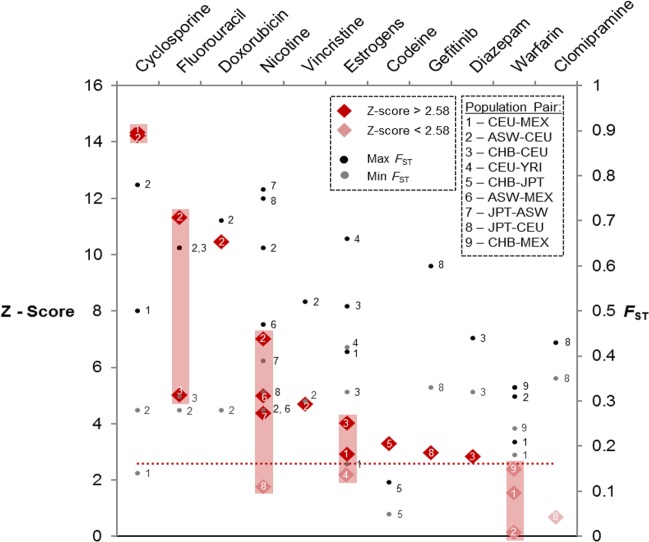
Evaluating relevance of pharmacogenomics workflow. Capturing real world reported drug response population differentiation cases: left-vertical axis shows the *Z*-scores of the respective drug in the specific population pair that is most similar to the population pair reported in the literature. Diamonds represent the specific drug *Z*-score in the specific population pair. Dark red diamond indicates *Z*-score > 2.58 while light red diamond indicates *Z*-score < 2.58. The red shaded area shows the difference between the highest and lowest *Z*-scores for that drug. Right-vertical axis shows the highest and lowest *F*_ST_ scores of the top pf-pdSNPs in the specific drug pathway and they are represented by black and grey dots, respectively. Numbers correspond to the specific population pairs used for analyses and reported in literature

Figure 3, shows the *Z*-scores for the enrichment of the pf-pdGenes, as well as the maximum and minimum SNP *F*_ST_ scores associated with these 11 well-known drugs/compounds. With the exception of clomipramine and warfarin, the *Z*-scores of all the other drugs were above the stringent threshold of 2.58 in at least one of the reported specific population pairs. In total, this approach was able to detect ~82% of the reported population differentiation cases, since nine of 11 drugs reported are shown to be enriched by genes exhibiting population differentiation (*Z*-score > 2.58). Interestingly, the *Z*-scores for clomipramine in 14 population-pair combinations were statistically significant (*Z* > 2.58) suggesting that this drug exhibits significant differentiation in these population pairs. However, the difference was not statistically significant in the population pair reported in the literature (JPT-CEU). In the case of warfarin, both VKORC1 and CYP2C9 genes, which are known to be pertinent to warfarin response, were also pf-pdGenes in this study (Supplementary Fig. 3**)**. However, as there was a large number of other genes in this pathway, the effects of these two pf-pdGenes were diluted, causing warfarin to fail the enrichment analysis.

### Drug/disease classes associated with population-differentiated genes

**Fig. 4:**
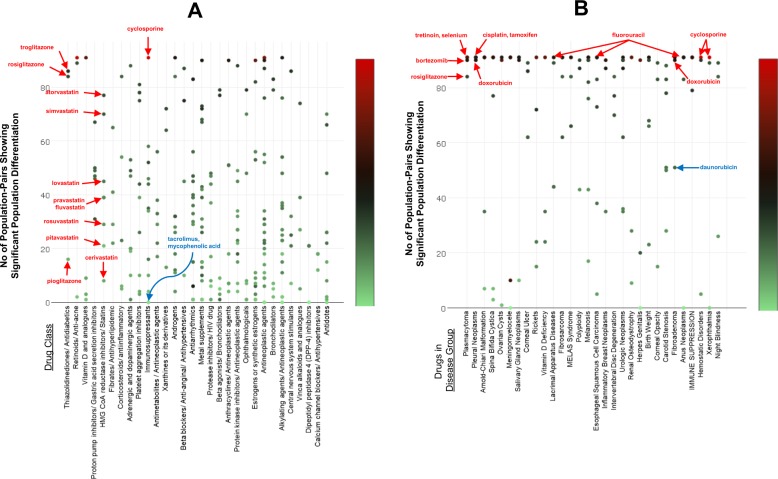
Drug/disease classes associated with population differentiated genes. **a** The number of population-pairs showing significant population genetic differentiation in drugs across different drug class. Each dot represent a drug under the respective drug class. The *y*-axis indicates the number of population-pairs showing significant population differentiation while the *x*-axis represents the top 30 drug classes. **b** The number of population-pairs showing significant population genetic differentiation in drugs across different disease/condition groups. Each dot represent a drug under the respective disease group. The *y*-axis indicates the number of population-pairs showing significant population differentiation and the *x*-axis shows the top 30 disease/condition groups

Figure 4a, shows the top 30 drug classes (including antidiabetics, statins, anti-inflammatory, immunosuppression, and antineoplastic drugs), with constituent drugs that are genetically differentiated in the most number of population-pairs. Within each drug class, there are drugs with very high *Z*-scores across many populations (e.g., cyclosporine), as well as drugs with lower *Z*-scores across fewer populations (tacrolimus, mycophenolic acid). Likewise, Fig. 4b, shows the top 30 disease/condition groups containing drugs indicated for its treatment, which are highly differentiated in the most number of population-pairs. Again, within each treatment class, there are drugs with very high *Z*-scores across many populations, as well as drugs with lower *Z*-scores across fewer populations.

### Drugs with existing PGx warning labels or ADR reports are associated with the number of significantly enriched population pairs

**Fig. 5:**
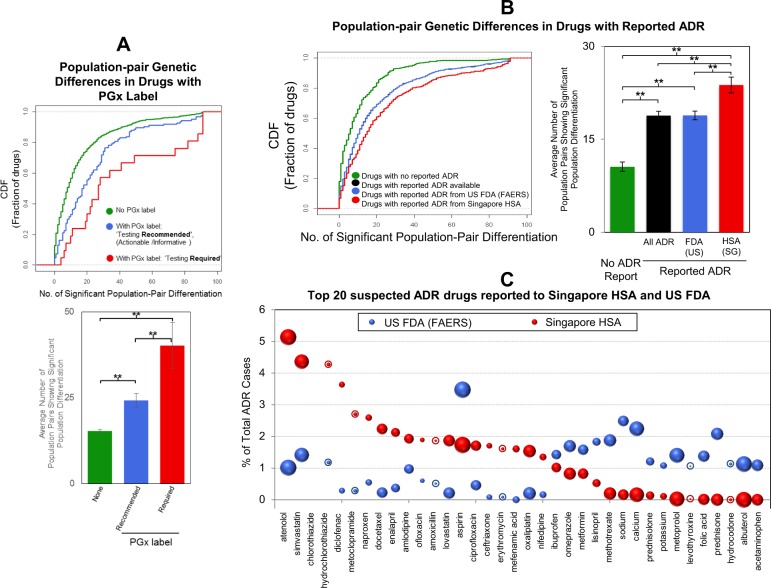
Population-pair genetic differences across drugs with existing PGx warning labels or suspected to cause adverse drug reactions (ADRs). **a** Relevance of data analytics approach for drugs with existing PGx label as shown by the cumulative distribution function (CDF) of the number of significant population-pair against the fraction of drugs. Drugs with PGx label indicating ‘genetic testing required’ (red line) and drugs with PGx label indicating ‘testing recommended’ (including ‘actionable’ and ‘informative’ PGx) (blue line) are compared with drugs with no existing PGx label (green line). The average of the number of population pairs showing significant population genetic differentiation across the three group of drugs is shown by the bar chart below (**P < 0.01). Error bars represent the standard error of the mean. **b** CDF of the number of significant population-pair differences in drugs with reported ADR (black line, underneath blue line), as well as those with ADR reported by the US FDA FAERS database (blue line) and the Singapore HSA database (red line) were compared against drugs with no ADR report (green line). The average of the number of population pairs showing significant population genetic differentiation across drugs belonging to the four groups is shown by the bar chart (**P < 0.01). Error bars represent the standard error of the mean. **c** Percentage of the total ADR cases of the top 20 ADR drugs reported to Singapore’s HSA (Red Balls) and USA FDA FAERS (Blue Balls). Size of balls denotes the total number of population pairs showing significant population genetic differentiation in the drugs. Balls with ‘halo’ represent ADR drugs that are not significantly population-differentiated in ≥10 population pairs

To further assess the practical relevance of our approach, drugs with existing PGx warning labels issued by either the US FDA (also commonly referred to as black box warning), European EMA, Japanese PMDA, or Canadian HCSC, were examined to determine if they are likely to be enriched by genes exhibiting population differences. From the 1512 FDA-approved drugs examined, 150 drugs had PGx labels (mainly with information associated with germline variants) issued by at least one of the authorities. Twenty-three of these drugs had the strongest warning of ‘genetic testing required’, while the other 128 drugs had a milder warning of ‘genetic testing recommended’, ‘actionable PGx’, or ‘informative PGx’ labels. As shown from the CDF plot in Fig. 5a (top), 50% of drugs with no PGx label (green line) had ten or less significantly differentiated population pairs. However, 50% of drugs with labels indicating testing recommended, actionable or informative (blue line) had 20 or less significantly differentiated population pairs, and this number was even higher for drugs with the ‘genetic testing required’ label (red line, 30 or less). This suggests that the number of differentiated population pairs is positively associated with the severity of the PGx label. Furthermore, the average number of population pairs with genomic differentiation in the three groups were found to be significantly different from each other (*P* < 0.01, Student's *t*-test) (Fig. 5a, bottom) with the ‘genetic testing required’ group having the highest average number.`

We further explored if drugs with reported suspected ADR were also associated with genes carrying significant population-differentiated variants. Of the 1512 FDA-approved drugs/compounds examined, ~70% (1058) had at least one ADR report in the database. As shown in Fig. 5b (left), for the same fraction of drugs with no reported ADR (green line),there were fewer population-pairs having significant genomic differentiation compared with those with suspected ADR reports (red line or blue line). Drugs with ADR reports from Singapore’s HSA (red bar) had a significantly higher average number of population-pairs with population genomic differentiation than those reported by the US-FAERS (blue bar) (*P* < 0.01, Student's *t*-test) (Fig. 5**b**, right).

The ADR drug profiles in Singapore are different from those in the USA (Fig. 5c). For example, the frequency of ADR cases due to atenolol was ~5% in Singapore (red ball), but only ~1% in the USA (blue ball). On the other hand, frequency of ADR cases due to aspirin was 3.5% (blue dot) in the USA, but only 1.7% in Singapore (red dot). Approximately 78% (28/36) of these commonly reported ADR drugs were linked to significant genomic differentiation (*Z* > 2.58) in ten or more population pairs (Fig. 5c, without halo). Twelve of the top 20 ADR drugs (Supplementary Table 6) were also in the top 30 drug classes with the most number of population pair differences while five of the top 20 ADR drugs (Supplementary Table 7) were in the top 30 disease/condition categories.

## Discussion

The availability of comprehensive genomic and drug–gene knowledge databases, coupled with the power of ‘big data’ and deep analytics, can facilitate the development of novel pharmacogenomics workflows to identify drugs that are significantly associated with genes carrying population differentiated variants. Here, we present a novel approach at identifying such drugs. By examining the genomes from 14 world populations, pfSNPs in drug pathways that display significant population differences in their allele frequencies were identified. To our knowledge, this is the first attempt at developing a large database of genomic SNPs, which are not only potentially functional but also display significant population differentiation (pf-pdSNPs). Furthermore, a unique database of drugs with genes that are differentiated between specific population pairs was also developed. These data may facilitate the design of genetic assays and novel SNPs chip to screen individuals carrying variant(s) that may influence the gene function, and hence alter the drug response. Such information may also provide insights into the molecular mechanism of the specific drug pathway responsible for modulating population differences in response.

To examine the translational application of our approach, 1512 FDA-approved drugs/compounds (of 10,902 drugs with available gene information) were further analyzed. Approximately 230 of these drugs were either lacking any pf-pdSNPs, or not enriched with pf-pdGenes. This suggests that these drugs are not associated with genes that are significantly population differentiated, and are likely to be similarly effective for any population. Other drugs are observed to be associated with genes carrying SNPs that are significantly differentiated in at least one population pair, with 13 drugs, including immunosuppressants and a few anticancer drugs (cisplatin, fluorouracil, tamoxifen, and decitabine), being associated with significantly population-differentiated genes in all 91-population pairs tested. As such, development of custom pf-pdSNP genotyping panel and/or PGx resources may guide clinicians in their choice of drugs to treat patients from specific ethnic populations. In cases where the drug is enriched with population-differentiated associated genes, one could consider substituting the drug with an alternative drug from a similar class that is less population differentiated especially between the population of the patient and the population where the drug was trialled. For example, cyclosporine was predicted in this study to be significantly enriched with pf-pdSNPs in all 91 population-pairs comparison (Fig. 2b), and was also previously reported in the literature to show significant differences in response between different population pairs (e.g., Europeans versus Africans) (Fig. 3). Hence, instead of prescribing cyclosporine, one can perhaps refer to Fig. 4 to identify alternative drugs (e.g., tacrolimus) in the same drug class that are less population differentiated to prescribe. The suggestion of prescribing tacrolimus instead of cyclosporine is consistent with previous literature reporting that cyclosporine is associated with greater nephrotoxicity than tacrolimus [60]. In addition, the role of population-differentiated variations of genes in the pathway of the drug could also be further elucidated, and this would help facilitate better understanding of how these genes modulate drug efficacy or toxicity.

Another important finding from this study is that a significantly greater proportion of drugs with mandatory genetic testing requirements were associated with genes that are differentiated in more population-pairs than those with milder or no PGx recommendations. Furthermore, drugs suspected of causing ADR in Singapore had significantly higher number of population pairs with population-differentiated genes than those reported to cause ADR in the USA or drugs not reported to cause ADR. This is consistent with our hypothesis that Singapore, which imports drugs from the US, has different ethnic groups with different genetic backgrounds and drug responses that may result in a higher risk of ADR. Notably, the ADR incidence profiles of drugs and drug classes causing Stevens Johnson syndrome (SJS) and toxic epidermal necrolysis (TEN), two severe and potentially life threatening ADRs, were found to be different in Singapore (HSA) compared with USA (FAERS) (Supplementary Fig. 4). The top drugs suspected to cause SJS/TEN were significantly enriched with pf-pdGenes in many population pairs.

Taken together, our results suggest that drugs with ADR are associated with population-differentiated genes, and that the number of population pairs that are significantly enriched in a drug is a good indicator of its potential to cause adverse reactions. There are however several caveats to using the ADR reports, especially those from Singapore’s HSA adverse events monitoring program. These reports are likely to be biased as they are based on limited or incomplete data, as well as a variable degree of both underreporting and pattern of reported drug usage. In addition, ADR reporting frequency varies in different countries, which may account for some of the differences observed. Nevertheless, our finding that there is an association between ADR incidence/severity of PGx warning labels and the number of enriched population pairs provides further evidence for the usefulness of the population pair data as well as for our overall approach.

The real-world relevance of our deep analytics approach was evident from its ability to detect significant genomic population differentiation in >80% of the 11 drugs previously reported to show population differences in response. While this is a good level of accuracy, there are still some areas that could be improved upon. Currently, the algorithm’s strength lies in its ability to generate a drug’s enrichment *Z*-score by utilizing information from multiple genes or variants. However, in the case of warfarin, although the genes (VKORC1, CYP2C9) involved in warfarin response were, indeed classified as pf-pdGenes, significant enrichment of pf-pdGenes was not detected. The reason is because enrichment was calculated by considering these two genes as well as all the other genes in the warfarin pathway. This pool of other drug-response genes could possibly dilute the effect of the two reported genes associated with warfarin. Future improvements to our algorithm could include a feature that puts additional weights on well-validated single genes or variants associated with population difference in specific drugs. Alternatively, machine learning or artificial intelligence methods could be used to improve the predictive ability of the algorithm. In addition, factors such as drug interactions, patient’s age, gender, lifestyle, and environmental variables could be incorporated into the model.

The pipeline can also be adapted to include newly developed drugs if the drug–genes relationship is known, or if this relationship can be inferred from other parameters such as structural similarity to existing drugs. As knowledge about key polymorphisms driving drug response increases, it is likely that the accuracy of the algorithm would also increase. All these features would then be coupled with a user-friendly interface to facilitate the querying of drugs, drug class, population-pair, disease category, genes, or SNPs and will eventually provide a useful resource for evaluating genomic population-difference status, as well as to provide candidate molecules and genes to include in a novel PGx screening assay.

In conclusion, our approach represents a significant advancement towards the utilization of big-data genomics in precision medicine. The population-pair specific information generated from this study can help facilitate decision-making by relevant authorities across the globe. These include decisions about whether a specific drug that has been tested to be effective in one population should be given to another population without testing, whether the drug should also be trialled in the other population, or whether a genetic test targeting the pf-pdSNPs should be conducted before the drug is given. Furthermore, alternative drugs that do not exhibit population differentiation can be proposed, and medication from the WHO Essential Medicines List can also be selected based on the drug’s population genetics profile. By leveraging on this technology, it is hoped that many of these scenarios can be realized in the near future.

## Supplementary information

Supplementary Material

## Data Availability

Code is available upon request.
