# Supplementary material for: Towards precision medicine: interrogating the human genome to identify drug pathways associated with potentially functional, population-differentiated polymorphisms
Source: Pharmacogenomics J. 2019 Oct 3;19(6):516–27. doi: 10.1038/s41397-019-0096-y (PMC6867962; doi:10.1038/s41397-019-0096-y)
Supplement: Supplementary file 1 — Supplementary Material [file 41397_2019_96_MOESM1_ESM.docx]

**Supplementary Information**

**Towards Precision Medicine: Interrogating the Human Genome to Identify Drug Pathways Associated with Potentially Functional, Population-Differentiated Polymorphisms**

Maulana Bachtiar, Brandon Nick Sern Ooi, Jingbo Wang, Jin Yu, Tin Wee Tan,

Samuel S. Chong, and Caroline G. L. Lee*

*Corresponding author’s e-mail: [bchleec@nus.edu.sg](mailto:bchleec@nus.edu.sg)

This file includes:

**Supplementary Figure 1.** Databases employed to associate drugs with genes.

**Supplementary Figure 2.** Association of drugs with genes carrying pdSNPs and pf-pdSNPs.

**Supplementary Figure 3.** Distribution of FST scores for warfarin SNPs in the 91 population pairs.

**Supplementary Figure 4**. SJS/TEN (Stevens-Johnson syndrome/Toxic Epidermal Necrolysis) cases reported to HSA (Singapore) or FAERS (USA).

**Supplementary Table 1.** Algorithms predicting functional significance of SNPs from the pfSNP resource (http://pfs.nus.edu.sg). (Adapted from (Wang, Ronaghi, Chong & Lee, 2011))

**Supplementary Table 2.** Explanation of abbreviations.

**Supplementary Table 3.** Several examples of literature evidence associating genes with the drug statin as annotated by the Comparative Toxicogenomics Database (CTD).

**Supplementary Table 4.** References relevant for the drugs that were reported to be population differentiated.

**Supplementary Table 5.** FDA-approved drugs/compounds that are NOT associated with genes carrying significantly population-differentiated SNPs (pdGenes)

**Supplementary Table 6.** Top ADR drugs that belong to the top 30 drug classes

**Supplementary Table 7.** Top ADR drugs that belong to the top 30 disease/condition categories

## SUPPLEMENTAL FIGURES

**
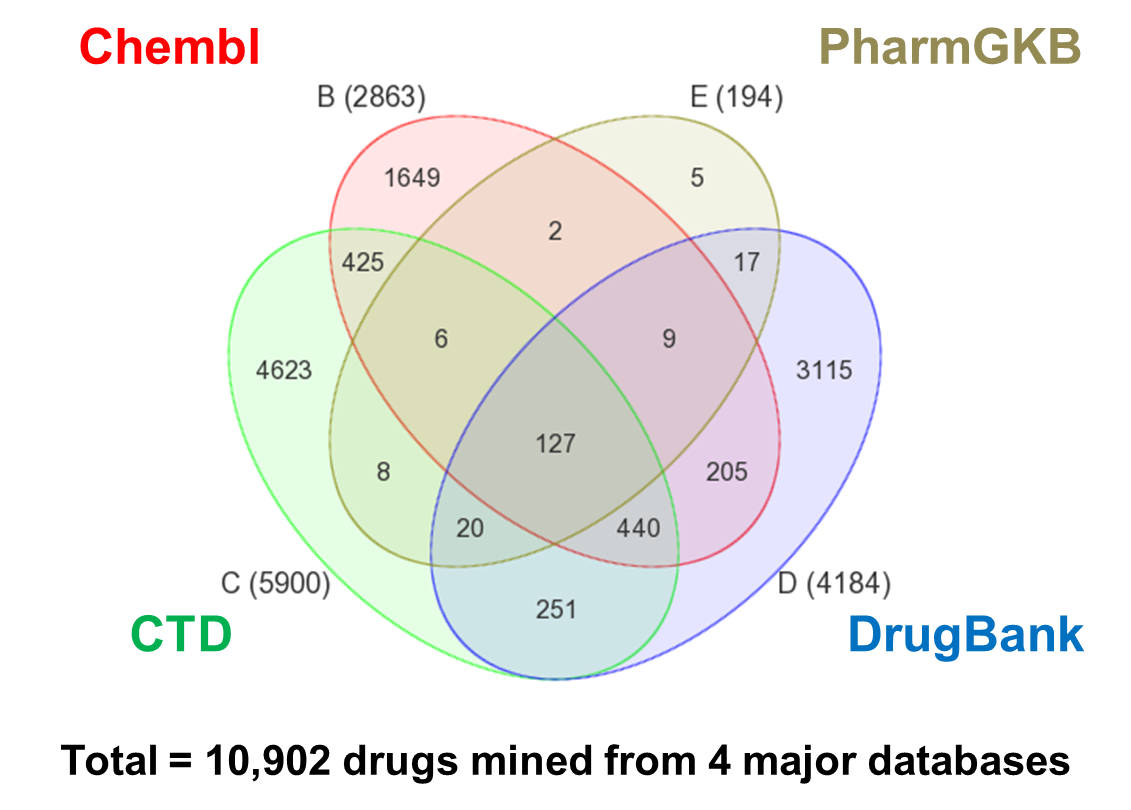
**

### Supplementary Figure 1. Databases employed to associate drugs with genes.

Number in the Venn Diagram represents the number of drugs from the various databases that could be associated with genes.


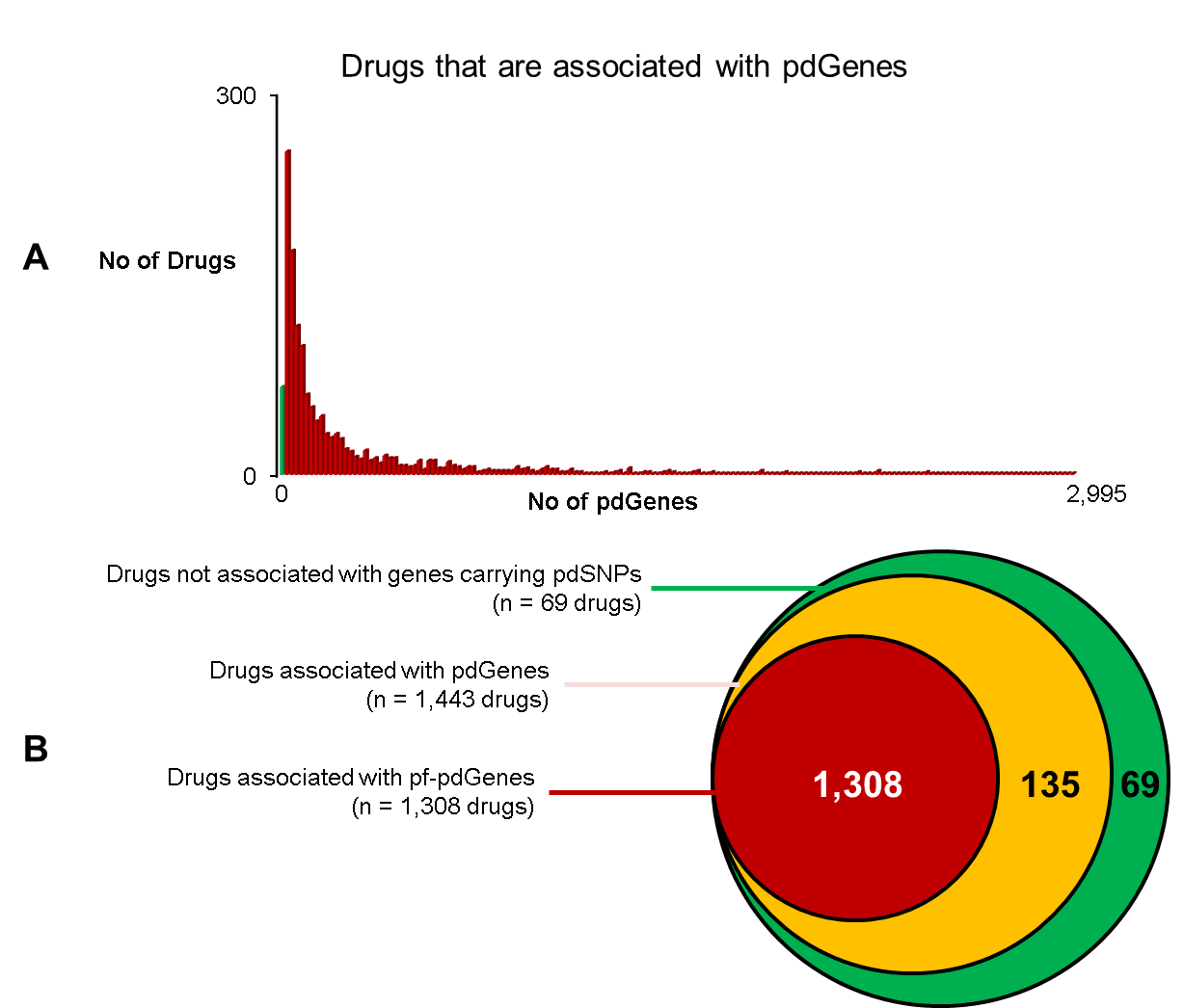


### Supplementary Figure 2. Association of drugs with genes carrying pdSNPs and pf-pdSNPs.

(A) The distribution of the number of pdGenes carrying population-differentiated SNPs (pdSNPs) that are associated with FDA-approved drugs. The y-axis indicate the number of drugs that are associated with the different number of pdGenes (x-axis). (B) Out of a total of 1,512 FDA-approved drugs/compounds, 1,443 are associated with pdGenes, of which 1,308 drugs are linked to pf-pdGenes carrying potentially functional-population differentiated SNPs (pf-pdSNPs). On the other hand, 69 drugs are not associated with such genes (also reflected by the green bar in A).

### Supplementary Figure 3. Distribution of FST scores for warfarin SNPs in the 91 population pairs.

SNPs that are population differentiated are positioned within the top 1% of the respective population pair comparison (outside of the blue shade).

**
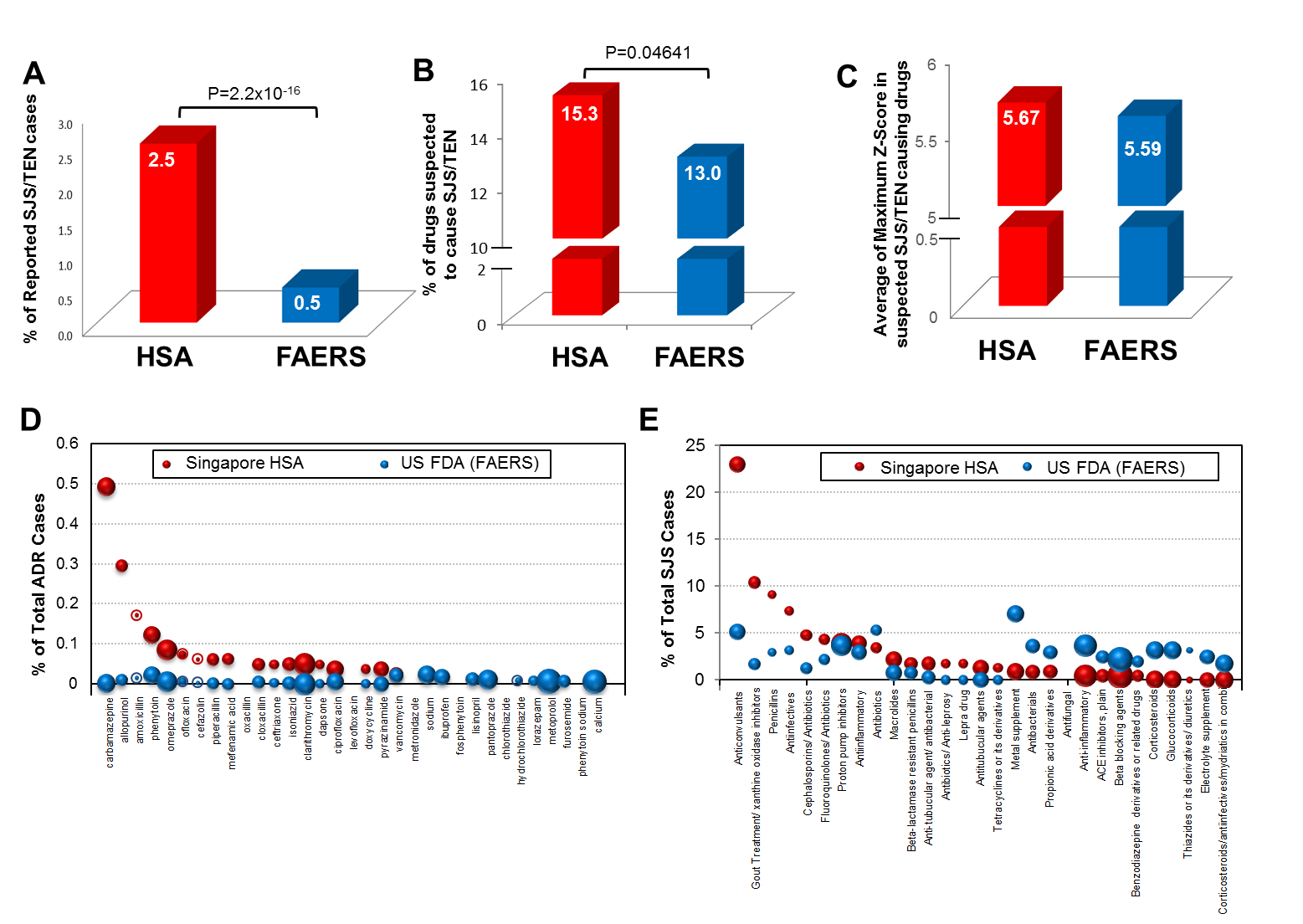
**

### Supplementary Figure 4. SJS/TEN (Stevens-Johnson syndrome/Toxic Epidermal Necrolysis) cases reported to HSA (Singapore) or FAERS (USA).

1. Percentage of reported ADR cases that are SJS/TEN in Singapore (HSA) or USA (FAERS).
2. Percentage of ADR-related drugs that are suspected to cause SJS/TEN.
3. Average value of the maximum Z-score in suspected SJS/TEN Drugs.
4. Percentage of the total ADR cases of the top 20 ADR drugs that are suspected to cause SJS/TEN reported to Singapore’s HSA (Red Balls) and USA FAERS (Blue Balls). Size of balls denotes the total number of population pairs showing significant population differentiation in the drugs. ADR drugs not found to be population-differentiated in 10 or more population pairs are indicated by balls with halo.
5. Percentage of the total suspected SJS/TEN Drugs in the specific drug class. Red Balls: Singapore’s HSA; Blue Balls: USA FAERS. Size of balls denotes the proportion between the observed total number of population pairs showing significant population differentiation over the total number of maximum possible number of population pairs involving drugs within the respective class.

A severe and potentially life-threatening ADR is Stevens Johnson syndrome (SJS) and toxic epidermal necrolysis (TEN). Previous studies reported that the proportion of SJS/TEN cases due to carbamazepine and phenytoin drugs were significantly higher in South East Asia (26%) than in the European populations (12%) (Lee, Martanto & Thirumoorthy, 2013; Sassolas et al., 2010). From our data, we also found a significantly greater percentage of ADR cases that reported SJS/TEN in Singapore (2.5%) compared to the USA (0.5%) (**Supplementary Figure 3 a**) as well as a significantly greatly proportion of ADR drugs that are suspected to cause SJS/TEN in Singapore (15.3%) compared to the USA (13%) (**Supplementary Figure 3 b**). Notably, there is a relatively higher average number of the maximum Z-score in suspected SJS/TEN causing drugs in Singapore (5.67) compared to the US (5.59) albeit no statistical significance was achieved (**Supplementary Figure 3 c**).

The profile of drugs/drug classes that are suspected to cause SJS/TEN is also very different in Singapore compared to the USA. For example, carbamazepine and allopurinol are the top two reported drugs that are most frequently suspected to cause SJS/TEN in Singapore accounting for 0.49% and 0.3% of all ADR cases in Singapore, respectively (**Supplementary Figure 3 d**). However, in the USA, these two drugs accounted for only 0.001% and 0.01%, respectively of all the FAERS ADR cases and are not amongst top SJS/TEN drugs in the USA. Similarly, while the anticonvulsants drug class is the most significant drug class suspected to cause SJS/TEN in Singapore (22.94% of SJS/TEN cases), this drug class constitutes only 5.1% of all drugs suspected to cause SJS/TEN in the US (**Supplementary Figure 3 e**). Metal supplements is the most significant drug class suspected to cause SJS/TEN in the USA (7.04%) but constitutes only 0.87% in Singapore. Notably, all of the top drug classes consist of one or more drugs that are significantly enriched by genes exhibiting population differences.

## SUPPLEMENTARY REFERENCES

Lee HY, Martanto W, & Thirumoorthy T (2013). Epidemiology of StevenseJohnson syndrome and toxic epidermal necrolysis in Southeast Asia. DERMATOLOGICA SINICA 31**:** 217-220.

Sassolas B, Haddad C, Mockenhaupt M, Dunant A, Liss Y, Bork K*, et al.* (2010). ALDEN, an algorithm for assessment of drug causality in Stevens-Johnson Syndrome and toxic epidermal necrolysis: comparison with case-control analysis. Clin Pharmacol Ther 88**:** 60-68.

## SUPPLEMENTARY TABLES

**Supplementary Table 1. Algorithms predicting functional significance of SNPs from the pfSNP resource (http://pfs.nus.edu.sg). (Adapted from (Wang, Ronaghi, Chong & Lee, 2011))**

| **Type** | **Subtype** | **SNP Functional Category** | **Algorithms** |
| --- | --- | --- | --- |
| Coding SNPs | All | Protein domain/functional region | Interpro Scan, Tm HMM (Transmembrane domain), NetOGlyc (O Glycosylation site), NetNGlyc (N Glycosylation site), NetPhos (Phosphorylation site) (Zdobnov & Apweiler, 2001) |
|  |  | Nonsense-mediated decay (NMD) | Nonsense Mediated Decay (Nagy & Maquat, 1998) |
|  |  | Exonic splice enhancer/silencer (ESE/ESS) | RESCUE-ESE (Fairbrother, Yeh, Sharp & Burge, 2002), Enrichment detection (Wang, Rolish, Yeo, Tung, Mawson & Burge, 2004; Zhang & Chasin, 2004), Hex mer library screening (Zhang & Chasin, 2004), Experimentally validated ESE/ESS (Zhang & Chasin, 2004) |
|  | Synonymous SNPs | Difference in Codon usage | Codon usage differences (http://www.kazusa.or.jp/codon/) |
|  | Non-synonymous | Predicted to be Deleterious | Polyphen (Ramensky, Bork & Sunyaev, 2002), SNPs3D (Yue, Melamud & Moult, 2006), LS-SNP (Karchin et al., 2005), Panther-SNP (Thomas et al., 2003) |
| Non-coding SNPs | Promoter / 5'UTR | Transcription factor binding site (TFBS) | TransFac (http://www.biobase-international.com/pages/index.php?id 5 transfac) |
|  | Intron | Intronic splice regulatory element (ISRE) | Conserved intronic sequence near splice site (Yeo, Van Nostrand & Liang, 2007) |
|  |  | Alter Splice Sites | Alter splice site sequence (Sahashi et al., 2007) |
|  | 3'UTR | miRNA binding sites | MiRanda (Saunders, Liang & Li, 2007), PolymiRTS Database (Bao et al., 2007), Patrocles Database (Hiard, Charlier, Coppieters, Georges & Baurain, 2010) |
|  |  | 3’ UTR conserved regions | Conserved regions in 3' UTR (Xie et al., 2005) |

References for Supplementary Table 2:

Bao L, Zhou M, Wu L, Lu L, Goldowitz D, Williams RW, et al. (2007). PolymiRTS Database: linking polymorphisms in microRNA target sites with complex traits. Nucleic Acids Res 35: D51-54.

Fairbrother WG, Yeh RF, Sharp PA, & Burge CB (2002). Predictive identification of exonic splicing enhancers in human genes. Science 297: 1007-1013.

Hiard S, Charlier C, Coppieters W, Georges M, & Baurain D (2010). Patrocles: a database of polymorphic miRNA-mediated gene regulation in vertebrates. Nucleic Acids Res 38: D640-651.

Karchin R, Diekhans M, Kelly L, Thomas DJ, Pieper U, Eswar N, et al. (2005). LS-SNP: large-scale annotation of coding non-synonymous SNPs based on multiple information sources. Bioinformatics 21: 2814-2820.

Nagy E, & Maquat LE (1998). A rule for termination-codon position within intron-containing genes: when nonsense affects RNA abundance. Trends Biochem Sci 23: 198-199.

Ramensky V, Bork P, & Sunyaev S (2002). Human non-synonymous SNPs: server and survey. Nucleic Acids Res 30: 3894-3900.

Sahashi K, Masuda A, Matsuura T, Shinmi J, Zhang Z, Takeshima Y, et al. (2007). In vitro and in silico analysis reveals an efficient algorithm to predict the splicing consequences of mutations at the 5' splice sites. Nucleic Acids Res 35: 5995-6003.

Saunders MA, Liang H, & Li WH (2007). Human polymorphism at microRNAs and microRNA target sites. Proc Natl Acad Sci U S A 104: 3300-3305.

Thomas PD, Kejariwal A, Campbell MJ, Mi H, Diemer K, Guo N, et al. (2003). PANTHER: a browsable database of gene products organized by biological function, using curated protein family and subfamily classification. Nucleic Acids Res 31: 334-341.

Wang Z, Rolish ME, Yeo G, Tung V, Mawson M, & Burge CB (2004). Systematic identification and analysis of exonic splicing silencers. Cell 119: 831-845.

Xie X, Lu J, Kulbokas EJ, Golub TR, Mootha V, Lindblad-Toh K, et al. (2005). Systematic discovery of regulatory motifs in human promoters and 3' UTRs by comparison of several mammals. Nature 434: 338-345.

Yeo GW, Van Nostrand EL, & Liang TY (2007). Discovery and analysis of evolutionarily conserved intronic splicing regulatory elements. PLoS Genet 3: e85.

Yue P, Melamud E, & Moult J (2006). SNPs3D: candidate gene and SNP selection for association studies. BMC Bioinformatics 7: 166.

Zdobnov EM, & Apweiler R (2001). InterProScan--an integration platform for the signature-recognition methods in InterPro. Bioinformatics 17: 847-848.

Zhang XH, & Chasin LA (2004). Computational definition of sequence motifs governing constitutive exon splicing. Genes Dev 18: 1241-1250.

**Supplementary Table 2. Explanation of abbreviations**

**Supplementary Table 3. Examples of literature evidence associating genes with the drug statin as annotated by the Comparative Toxicogenomics Database (CTD).**

| **Gene Symbol** | **Gene Name** | **Summary** | **References** |
| --- | --- | --- | --- |
| **APOE** | **apolipoprotein E** | APOE affects the susceptibility to Simvastatin | Wang YX, et al. |
|  |  |  | Anti-atherosclerotic effect of simvastatin depends on the presence of apolipoprotein E. |
|  |  |  | ***Atherosclerosis. 2002 May;162(1):23-31***. |
|  |  | APOE polymorphism affects the susceptibility to Simvastatin | Ye P, et al. |
|  |  |  | The influence of apolipoprotein B and E gene polymorphisms on the response to simvastatin therapy in patients with hyperlipidemia. |
|  |  |  | ***Chin Med Sci J. 2003 Mar;18(1):9-13***. \| |
|  |  |  | Vohl MC, et al. |
|  |  |  | Influence of LDL receptor gene mutation and apo E polymorphism on lipoprotein response to simvastatin treatment among adolescents with heterozygous familial hypercholesterolemia. |
|  |  |  | ***Atherosclerosis. 2002 Feb;160(2):361-8***. |
|  |  | Polystyrenes analog inhibits the reaction [Simvastatin results in decreased expression of APOE protein] | Mitchell A, et al. |
|  |  |  | The effect of the HMG-CoA reductase inhibitor simvastatin and of cholestyramine on hepatic apolipoprotein mRNA levels in the rat. |
|  |  |  | ***Biochim Biophys Acta. 1993 Mar 17;1167(1):9-14***. |
|  |  | Simvastatin results in decreased expression of APOE | Dong W, et al. |
|  |  |  | Differential effects of simvastatin and pravastatin on expression of Alzheimer''s disease-related genes in human astrocytes and neuronal cells. |
|  |  |  | J ***Lipid Res. 2009 Oct;50(10):2095-102***. |
|  |  | Simvastatin results in decreased expression of APOE mRNA | Beverly BE, et al. |
|  |  |  | Simvastatin and dipentyl phthalate lower ex vivo testicular testosterone production and exhibit additive effects on testicular testosterone and gene expression via distinct mechanistic pathways in the fetal rat. |
|  |  |  | ***Toxicol Sci. 2014 Oct;141(2):524-37***.\| |
|  |  |  | Guan JZ, et al. |
|  |  |  | HMG-CoA reductase inhibitor, simvastatin improves reverse cholesterol transport in type 2 diabetic patients with hyperlipidemia. |
|  |  |  | ***J Atheroscler Thromb. 2008 Feb;15(1):20-5***. \| |
|  |  |  | Felgines C, et al. |
|  |  |  | Effect of simvastatin treatment on plasma apolipoproteins and hepatic apolipoprotein mRNA levels in the genetically hypercholesterolemic rat (RICO). |
|  |  |  | ***Life Sci. 1994;54(5):361-7***. \| |
|  |  |  | Mitchell A, et al. |
|  |  |  | The effect of the HMG-CoA reductase inhibitor simvastatin and of cholestyramine on hepatic apolipoprotein mRNA levels in the rat. |
|  |  |  | ***Biochim Biophys Acta. 1993 Mar 17;1167(1):9-14***. |
|  |  | Simvastatin results in decreased expression of APOE protein | Mitchell A, et al. |
|  |  |  | The effect of the HMG-CoA reductase inhibitor simvastatin and of cholestyramine on hepatic apolipoprotein mRNA levels in the rat. |
|  |  |  | ***Biochim Biophys Acta. 1993 Mar 17;1167(1):9-14***. |
| **MMP9** | **matrix metallopeptidase 9 (gelatinase B, 92kDa gelatinase, 92kDa type IV collagenase)** | farnesyl pyrophosphate inhibits the reaction [Simvastatin inhibits the reaction [Smoke analog results in increased secretion of MMP9 protein]] | Kim SE, et al. |
|  |  |  | Simvastatin inhibits induction of matrix metalloproteinase-9 in rat alveolar macrophages exposed to cigarette smoke extract. |
|  |  |  | ***Exp Mol Med. 2009 Apr 30;41(4):277-87***. |
|  |  | geranylgeranyl pyrophosphate inhibits the reaction [Simvastatin inhibits the reaction [[IL1A co-treated with PDGFB] results in increased secretion of MMP9 protein/mRNA]] | Turner NA, et al. |
|  |  | Mevalonic Acid inhibits the reaction [Simvastatin inhibits the reaction [[IL1A co-treated with PDGFB] results in increased secretion of MMP9 protein]] | Simvastatin inhibits MMP-9 secretion from human saphenous vein smooth muscle cells by inhibiting the RhoA/ROCK pathway and reducing MMP-9 mRNA levels. |
|  |  | Mevalonic Acid inhibits the reaction [Simvastatin inhibits the reaction [Tetradecanoylphorbol Acetate results in increased secretion of MMP9 protein]] | ***FASEB J. 2005 May;19(7):804-6***. |
|  |  | Simvastatin inhibits the reaction [Cholesterol, Dietary results in increased expression of MMP9 mRNA/protein] | Luo L, et al. |
|  |  |  | Hypercholesterolaemia induces early renal lesions characterized by upregulation of MMP-9 and iNOS and ET(A)R: alleviated by a dual endothelin receptor antagonist CPU0213 and simvastatin. |
|  |  |  | ***J Pharm Pharmacol. 2009 Jun;61(6):775-80***. |
|  |  | Simvastatin inhibits the reaction [[Dietary Fats co-treated with Cholesterol, Dietary] results in increased expression of MMP9 mRNA/protein] | Qin YW, et al. |
|  |  |  | Simvastatin inhibited cardiac hypertrophy and fibrosis in apolipoprotein E-deficient mice fed a "Western-style diet" by increasing PPAR α and γ expression and reducing TC, MMP-9, and Cat S levels. |
|  |  |  | ***Acta Pharmacol Sin. 2010 Oct;31(10):1350-8***. |
|  |  | Simvastatin inhibits the reaction [[IL1A co-treated with PDGFB] results in increased expression of MMP9 mRNA/protein] | Turner NA, et al. |
|  |  |  | Simvastatin inhibits MMP-9 secretion from human saphenous vein smooth muscle cells by inhibiting the RhoA/ROCK pathway and reducing MMP-9 mRNA levels. |
|  |  |  | ***FASEB J. 2005 May;19(7):804-6***. |
|  |  | Simvastatin inhibits the reaction [Lipopolysaccharides results in increased expression of MMP9 mRNA] | Sundararaj KP, et al. |
|  |  |  | Simvastatin suppresses LPS-induced MMP-1 expression in U937 mononuclear cells by inhibiting protein isoprenylation-mediated ERK activation. |
|  |  |  | ***J Leukoc Biol. 2008 Oct;84(4):1120-9***. |
|  |  | Simvastatin inhibits the reaction [Lipopolysaccharides results in increased expression of MMP9 protein] | Cuccurullo C, et al. |
|  |  |  | Suppression of RAGE as a basis of simvastatin-dependent plaque stabilization in type 2 diabetes. |
|  |  |  | ***Arterioscler Thromb Vasc Biol. 2006 Dec;26(12):2716-23***. |
|  |  | Simvastatin inhibits the reaction [Smoke analog results in increased activity of MMP9 protein] | Lee JH, et al. |
|  |  |  | Simvastatin inhibits cigarette smoking-induced emphysema and pulmonary hypertension in rat lungs. |
|  |  |  | ***Am J Respir Crit Care Med. 2005 Oct 15;172(8):987-93***. |
|  |  | Simvastatin inhibits the reaction [Smoke analog results in increased expression of MMP9 mRNA/protein] | Kim SE, et al. |
|  |  |  | Simvastatin inhibits induction of matrix metalloproteinase-9 in rat alveolar macrophages exposed to cigarette smoke extract. |
|  |  |  | ***Exp Mol Med. 2009 Apr 30;41(4):277-87***. |
|  |  | Simvastatin inhibits the reaction [Streptozocin results in increased expression of MMP9 mRNA] | Yao XM, et al. |
|  |  |  | Simvastatin protects diabetic rats against kidney injury through the suppression of renal matrix metalloproteinase-9 expression. |
|  |  |  | ***J Endocrinol Invest. 2010 May;33(5):292-6***. |
|  |  | Simvastatin inhibits the reaction [Tetradecanoylphorbol Acetate results in increased expression of MMP9 mRNA/protein] | Turner NA, et al. |
|  |  |  | Simvastatin inhibits MMP-9 secretion from human saphenous vein smooth muscle cells by inhibiting the RhoA/ROCK pathway and reducing MMP-9 mRNA levels. |
|  |  |  | ***FASEB J. 2005 May;19(7):804-6***. |
|  |  | Simvastatin inhibits the reaction [TNF protein results in increased expression of MMP9 protein] | Ahn KS, et al. |
|  |  |  | Simvastatin potentiates TNF-alpha-induced apoptosis through the down-regulation of NF-kappaB-dependent antiapoptotic gene products: role of IkappaBalpha kinase and TGF-beta-activated kinase-1. |
|  |  |  | ***J Immunol. 2007 Feb 15;178(4):2507-16***. |
|  |  | Simvastatin inhibits the reaction [TNF results in increased secretion of MMP9 protein/mRNA] | Turner NA, et al. |
|  |  |  | Simvastatin inhibits TNFalpha-induced invasion of human cardiac myofibroblasts via both MMP-9-dependent and -independent mechanisms. |
|  |  |  | ***J Mol Cell Cardiol. 2007 Aug;43(2):168-76***. \| |
|  |  |  | Porter KE, et al. |
|  |  |  | Tumor necrosis factor alpha induces human atrial myofibroblast proliferation, invasion and MMP-9 secretion: inhibition by simvastatin. |
|  |  |  | ***Cardiovasc Res. 2004 Dec 1;64(3):507-15***. |
|  |  | Simvastatin results in decreased activity of MMP9 protein | Cowled PA, et al. |
|  |  |  | Simvastatin plus nitric oxide synthase inhibition modulates remote organ damage following skeletal muscle ischemia-reperfusion injury. |
|  |  |  | ***J Invest Surg. 2008;21(3):119-26***. \| |
|  |  |  | Kim DY, et al. |
|  |  |  | Anti-inflammatory mechanism of simvastatin in mouse allergic asthma model. |
|  |  |  | ***Eur J Pharmacol. 2007 Feb 14;557(1):76-86***. \| |
|  |  |  | Thunyakitpisal PD, et al. |
|  |  |  | Simvastatin, an HMG-CoA reductase inhibitor, reduced the expression of matrix metalloproteinase-9 (Gelatinase B) in osteoblastic cells and HT1080 fibrosarcoma cells. |
|  |  |  | ***J Pharmacol Sci. 2004 Apr;94(4):403-9***. |
|  |  | Simvastatin results in decreased expression of and results in decreased activity of MMP9 protein | Aoki T, et al. |
|  |  |  | Simvastatin suppresses the progression of experimentally induced cerebral aneurysms in rats. |
|  |  |  | ***Stroke. 2008 Apr;39(4):1276-85***. \| |
|  |  |  | Kalyanasundaram A, et al. |
|  |  |  | Simvastatin suppresses experimental aortic aneurysm expansion. |
|  |  | Simvastatin results in decreased expression of MMP9 mRNA | ***J Vasc Surg. 2006 Jan;43(1):117-24***. |
| **LIF** | **leukemia inhibitory factor** | [Valsartan co-treated with Simvastatin] inhibits the reaction [Glucose promotes the reaction [AGT protein results in increased expression of LIF mRNA]] | Naito M, et al. |
|  |  |  | High ambient glucose augments angiotensin II-induced proinflammatory gene mRNA expression in human mesangial cells: effects of valsartan and simvastatin. |
|  |  |  | ***Am J Nephrol. 2009;30(2):99-111***. |
|  |  | beta-hydroxy simvastatin acid results in decreased expression of LIF mRNA | Tuomisto TT, et al. |
|  |  |  | Simvastatin has an anti-inflammatory effect on macrophages via upregulation of an atheroprotective transcription factor, Kruppel-like factor 2. |
|  |  |  | ***Cardiovasc Res. 2008 Apr 1;78(1):175-84***. |
| **TYMS** | **thymidylate synthetase** | Simvastatin results in decreased expression of TYMS mRNA | Takeda I, et al. |
|  |  |  | Simvastatin inactivates beta1-integrin and extracellular signal-related kinase signaling and inhibits cell proliferation in head and neck squamous cell carcinoma cells. |
|  |  | Simvastatin results in decreased expression of TYMS protein | ***Cancer Sci. 2007 Jun;98(6):890-9***. |

**Gene Symbol**

**Supplementary Table 4. References relevant for the drugs that were reported to be population differentiated.**

**Supplementary Table 5. FDA-approved drugs/compounds that are NOT associated with genes carrying significantly population-differentiated SNPs (pdGenes)**

| **No** | **Drug Name** |
| --- | --- |
| 1 | abarelix |
| 2 | acetates |
| 3 | acetyldigitoxin |
| 4 | acrivastine |
| 5 | ambenonium chloride |
| 6 | antazoline phosphate |
| 7 | arformoterol |
| 8 | atorvastatin calcium |
| 9 | azelastine hydrochloride |
| 10 | benzoate |
| 11 | benzylpenicilloyl polylysine |
| 12 | betamethasone dipropionate |
| 13 | bitolterol |
| 14 | bretylium |
| 15 | carboprost tromethamine |
| 16 | carglumic acid |
| 17 | clomipramine hydrochloride |
| 18 | colesevelam |
| 19 | cysteamine |
| 20 | dantrolene sodium |
| 21 | decamethonium bromide |
| 22 | degarelix |
| 23 | deslanoside |
| 24 | dibucaine hydrochloride |
| 25 | diethylamine |
| 26 | dronedarone |
| 27 | dyclonine hydrochloride |
| 28 | edrophonium chloride |
| 29 | eflornithine |
| 30 | fluvastatin sodium |
| 31 | formoterol fumarate |
| 32 | gemifloxacin |
| 33 | gonadorelin |
| 34 | halofantrine |
| 35 | hydrocortisone sodium succinate |
| 36 | hydroxyprogesterone caproate |
| 37 | hydroxystilbamidine isethionate |
| 38 | iodohippurate |
| 39 | isosorbide |
| 40 | lomefloxacin hydrochloride |
| 41 | methyprylon |
| 42 | metoprolol tartrate |
| 43 | mexiletine hydrochloride |
| 44 | nafarelin |
| 45 | nortriptyline hydrochloride |
| 46 | orphenadrine (citrate) |
| 47 | penicillin g potassium |
| 48 | pitavastatin calcium |
| 49 | polyestradiol phosphate |
| 50 | pravastatin sodium |
| 51 | procaine hydrochloride |
| 52 | propafenone hydrochloride |
| 53 | propranolol hydrochloride |
| 54 | protirelin |
| 55 | protriptyline hydrochloride |
| 56 | raltegravir |
| 57 | rivastigmine tartrate |
| 58 | sevelamer |
| 59 | sibutramine hydrochloride |
| 60 | silodosin |
| 61 | sodium nitroprusside |
| 62 | terbutaline sulfate |
| 63 | thiamylal sodium |
| 64 | thiopental sodium |
| 65 | thiothixene hydrochloride |
| 66 | trazodone hydrochloride |
| 67 | trimethaphan |
| 68 | trimipramine maleate |
| 69 | zanamivir |

**Supplementary Table 6. Top ADR drugs that belong to the top 30 drug classes**

**Supplementary Table 7. Top ADR drugs that belong to the top 30 disease/condition categories**
